# Detecting lateral gene transfers by statistical reconciliation of phylogenetic forests

**DOI:** 10.1186/1471-2105-11-324

**Published:** 2010-06-15

**Authors:** Sophie S Abby, Eric Tannier, Manolo Gouy, Vincent Daubin

**Affiliations:** 1 Université de Lyon; Université Lyon 1; CNRS; INRIA; UMR 5558, Laboratoire de Biométrie et Biologie Evolutive, 43 boulevard du 11 novembre 1918, F-69622 Villeurbanne, France

## Abstract

**Background:**

To understand the evolutionary role of Lateral Gene Transfer (LGT), accurate methods are needed to identify transferred genes and infer their timing of acquisition. Phylogenetic methods are particularly promising for this purpose, but the reconciliation of a gene tree with a reference (species) tree is computationally hard. In addition, the application of these methods to real data raises the problem of sorting out real and artifactual phylogenetic conflict.

**Results:**

We present Prunier, a new method for phylogenetic detection of LGT based on the search for a maximum statistical agreement forest (MSAF) between a gene tree and a reference tree. The program is flexible as it can use any definition of "agreement" among trees. We evaluate the performance of Prunier and two other programs (EEEP and RIATA-HGT) for their ability to detect transferred genes in realistic simulations where gene trees are reconstructed from sequences. Prunier proposes a single scenario that compares to the other methods in terms of sensitivity, but shows higher specificity. We show that LGT scenarios carry a strong signal about the position of the root of the species tree and could be used to identify the direction of evolutionary time on the species tree. We use Prunier on a biological dataset of 23 universal proteins and discuss their suitability for inferring the tree of life.

**Conclusions:**

The ability of Prunier to take into account branch support in the process of reconciliation allows a gain in complexity, in comparison to EEEP, and in accuracy in comparison to RIATA-HGT. Prunier's greedy algorithm proposes a single scenario of LGT for a gene family, but its quality always compares to the best solutions provided by the other algorithms. When the root position is uncertain in the species tree, Prunier is able to infer a scenario per root at a limited additional computational cost and can easily run on large datasets.

Prunier is implemented in C++, using the Bio++ library and the phylogeny program Treefinder. It is available at: http://pbil.univ-lyon1.fr/software/prunier

## Background

The systematic reconstruction of molecular phylogenies based on the diversity of genes found in complete genomes reveals an unforeseen degree of incongruence among gene trees. Different reasons, either biological or methodological, explain this diversity of patterns. First, evolutionary mechanisms such as gene duplication and loss, lateral gene transfer (LGT) and incomplete lineage sorting generate gene histories that deviate from that of species [1]. In unicellular organisms, and particularly Bacteria and Archaea, most of the real phylogenetic conflict is likely the result of LGT [2,3]. On the other hand, the reconstruction of gene histories based on sequence alignments is not trivial, and many artifacts are known which produce aberrant phylogenies due to stochastic effects or inadequate models of sequence evolution [4-7]. A challenge in the understanding of the patterns and processes of genome evolution is therefore to sort out these different sources of conflict.

The question of identifying LGTs based on phylogenies typically applies to the following data: first, a gene phylogeny, characterized by an unrooted tree topology, with branch lengths and statistical support for internal branches; and second, some knowledge of the evolutionary relationships among the organisms represented in this tree, ideally a rooted species phylogeny. Events of LGT can then be invoked to explain the topological discrepancies between the two trees. Among the various approaches that have been proposed to resolve the problem of tree reconciliation, the MAF (maximum agreement forest), and the closely related SPR (subtree pruning and regrafting) arguably represent the most appropriate models for the replacement of genes by LGT. The MAF problem consists in finding the smallest number of edges to cut in both trees in order to obtain two identical "forests" of rooted subtrees [8,9]. The SPR problem also corresponds to minimizing the number of subtrees to cut in a tree, but adds extra complexity by searching for the optimal place to regraft them. In the case of a rooted species tree, both approaches are equivalent to minimizing the number of LGTs that occurred in the gene family. These problems are known to be computationally difficult, but several algorithms have been proposed, notably to efficiently address the SPR problem. For instance, Than and Nakhleh have proposed a decomposition approach, implemented in the RIATA-HGT program [10,11], which identifies regions of the tree where the conflict can be resolved independently, and thus significantly reduces the complexity of the SPR reconciliation in many cases. EEEP also implements such a decomposition [12], and adds the possibility to restrict the type of SPR moves to those that immediately reduce the discordance among trees.

Obviously, an incorrectly reconstructed gene tree will lead to the inference of erroneous LGTs. It is therefore essential to take into account all information at hand on the reliability of the observed topological conflict, such as the length and support of all branches. Methods of LGT detection based on topological comparisons sometimes propose ways to incorporate the statistical information of the gene tree [11,13,12]. RIATA-HGT [11] first performs a purely topological reconciliation that proposes a collection of LGT scenarios. Each transfer is associated with a value which depends on the statistical support of the conflict it resolves in the gene tree, and the user can choose to ignore LGTs under a given threshold. EEEP [12] uses a different approach in which internal branches of the gene tree having a statistical support below a given threshold are collapsed *a priori*, before the trees are reconciled.

The two approaches described above have been implemented and tested on simulated datasets [11,12]. The main concern in both simulation setups was to evaluate time performance and the ability of the methods to recover the number of simulated LGTs in a gene tree. However, biologists are usually interested not only in the number of LGTs but more importantly in identifying the actual events of gene transfer, i.e. the exact set of species that are "misplaced" in the gene tree. Both approaches generally propose a number of evolutionary scenarios, but their accuracy has not been evaluated so far.

Here, we use simulations to explore the ability of different methods to detect events of transfers based on gene trees reconstructed from sequences. We introduce a greedy algorithm called "Prunier" [14], which uses information on topology, statistical support and branch lengths to quickly identify a maximum statistical agreement forest (MSAF) that corresponds to a most parsimonious scenario of transfer. Prunier uses a customizable statistical agreement function. Two implementations of this function were tested: a fast one based on branch support, and a more advanced one which uses the expected likelihood weights (ELW) test [15]. We show that working on reconstructed trees strongly affects the ability of different methods to identify transfer events. Although all methods can roughly estimate the number of LGTs that occurred in a gene history, the accuracy of proposed scenarios varies largely. In comparison with other methods, Prunier has lower false positive rate, which makes it a more accurate approach to detect LGTs in almost all simulated situations, especially for complicated gene histories. When tested on biological data of 23 universal proteins with hypothetical reference trees, Prunier revealed high rates of LGT, in particular in genes that are known to be prone to transfer. However, the degree of conflict in these genes raises concern on the approach used to reconstruct universal trees.

## Results

### Prunier Algorithm

#### Objectives

The phylogenetic detection of lateral gene transfers relies on differences between a gene tree *T*_*G*_, with branch lengths and support values, and a reference species tree *T*_*S *_on the same set of species *S*. Our method takes both trees as input. For clarity, we will suppose that the species tree is rooted, though the method offers the possibility of using an unrooted reference tree as input, with a reasonable increase in complexity (see *Material and Methods*). The input gene tree is always unrooted, and a byproduct of the procedure is to output a restrained set of root locations on the gene tree. Here we consider that topological differences between *T*_*S *_and *T*_*G *_can result either from LGT or from stochastic effects in the process of gene tree reconstruction. Therefore, an agreement function is needed to decide whether observed topological differences among trees are significant or not. Examples of such functions are maximum-likelihood tests comparing trees given a sequence alignment: KH (Kishino-Hasegawa [16], SH (Shimodaira-Hasegawa [17], AU (approximately unbiased [18] or ELW (expected likelihood weights [15] tests. Because these tests all take unrooted trees as input, we used additional criteria to be able to handle the disagreement for the position of the root when necessary (see *Material and Methods*). We also propose a faster alternative which simply considers the statistical support of internal branches. Two (sub-)trees are said to disagree if they fail the agreement function.

If a species tree and a gene tree disagree (based on the agreement function), the objective is to decompose them into a statistically agreeing forest. For a subset *S*_*i *_of the set of species *S*, we note *T*(*S*_*i*_) the subtree of *T *containing exactly *S*_*i *_where internal nodes of degree 2 are contracted. We aim at partitioning the set of species *S*into a minimum number of subsets *S*_*1 *_,...,*S*_*k *_such that for each subset *S*_*i*_, the two subtrees *T*_*S*_(*S*_*i*_) and *T*_*G*_(*S*_*i*_) agree, and all *T*_*S*_(*S*_*i*_) as well as all *T*_*G*_(*S*_*i*_) are disjoint. We call this partition a *maximal statistical agreement forest *(MSAF), in reference to the maximal agreement forest [8,9], which is the particular case where the agreement function gives a positive answer only if the two compared (sub-)trees are homomorphic (have the same topology).

Among *S*_*1 *_,...,*S*_*k *_, only one subset contains the root of the species tree. This is the non-transferred "backbone" of the tree as the root is its most ancient node and cannot acquire a gene from one of its descendants. The other *T*_*S*_(*S*_*i*_) of the forest are interpreted as LGTs that occurred in the last common ancestor of *S*_*i*_,. Their number (*k-1*) is the minimum number of transfer events that have to be invoked to explain the significant differences between the two trees.

#### Greedy Algorithm

**Figure 1:**
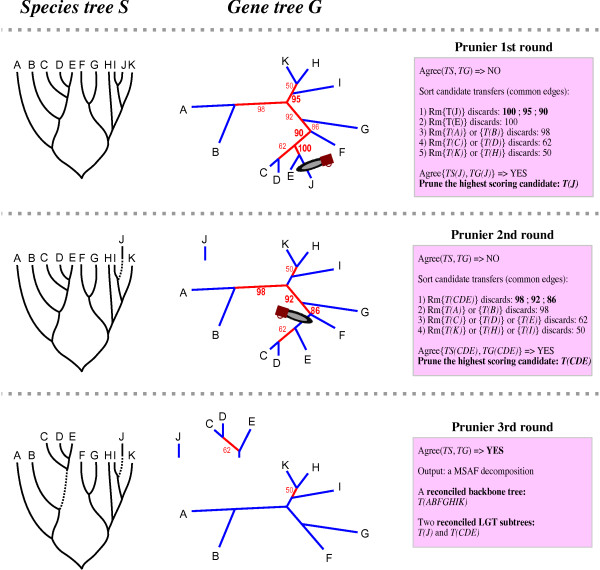
**Prunier algorithm: example of a Prunier run**. Example of reconciliation of an unrooted gene tree *T*_*G *_with a rooted species tree *T*_*S *_by searching for the maximum statistical agreement forest (MSAF). In the gene tree, blue branches are those common to the two trees whereas red branches indicate conflicting edges, *i.e.*, those found in *T*_*G *_but not in *T*_*S*_. Support values are shown for conflicting edges. In this example, two rounds are needed to reconcile the two trees. The agreement function "Agree" corresponds here to the "fast" version of Prunier: a gene (sub-)tree is considered in statistical agreement with the species (sub-)tree if no conflicting edge above 80 exists. At each round, clades corresponding to common edges (blue branches) are ranked by decreasing scores. This score reflects the conflict (combination of the support values) removed when the edge is cut (symbolized by "Rm{clade}"). The highest scoring subtree candidate to transfer is removed if it is in agreement with the species tree. The output of Prunier is a statistical agreement forest (SAF), composed of a reconciled backbone subtree (non-transferred sequences: {ABFGHIK}) and as many reconciled subtrees (transferred sequences) as lateral gene transfers (LGT). In this example, two LGT events are inferred: {J} and {CDE} have been transferred.

We use a greedy procedure to approach the maximum statistical agreement forest of two trees (see Fig. 1). Conflicting edges of the gene tree are those inducing bipartitions that are not in the species tree. A transfer event is characterized by a subtree *T*_*G*_(*S*_*i*_) that is in agreement with the corresponding *T*_*S*_(*S*_*i*_) but whose position is conflicting. In other words, an LGT results in a non conflicting edge whose removal decreases the conflict among trees. A non conflicting edge *e *cuts *T*_*S *_in two subtrees, one containing the root, the other defining a common clade *S*_*i*_. If *T*_*G*_(*S*_*i*_) and *T*_*s*_(*S*_*i*_) agree, the "conflict score" of *e *is a function (detailed in *Material and Methods*) of the number and support of the conflicting edges that are eliminated if *e *is removed from the tree. We consider the non conflicting edge with the highest conflict score as defining the most likely transfer. The procedure (Fig. 1) can be briefly described as follows:

• While the gene tree and species tree disagree:

▪ Remove the edge defining a statistically agreeing common clade with maximal conflict score

Often, several edges have the same conflict score. In this case, a different criterion is used to choose what edge to cut among top rating edges. We first use the alignment of the gene family to estimate branch lengths on both the gene and reference trees using Treefinder [19]. Then, for each candidate edge, we compute the difference between its lengths in both trees and cut the one with the highest difference. We hence remove the branch that is most affected when constrained to its reference position. In practice, branch lengths are estimated only once. This step appears to be necessary in most gene families and it accounts for most of the computing time in the "fast" implementation of Prunier.

Because only non conflicting edges are removed, the procedure always produces two subtrees, one of which is in agreement by construction, and the other (which contains the root) can be used recursively as an input of the function. Eventually, a statistical agreement forest is reached and each component of this forest which does not contain the root of T_G _is interpreted as originating from a transfer event. A scenario of transfer can readily be constructed from the comparison of T_G _with the forest.

The algorithm and its implementation are fully detailed in the *Material and Methods *section. In particular, the definition of the agreement function, conflict score and the position of the root are discussed.

### Accuracy on simulated LGTs

We used the procedure described in [20] (see *Material and Methods *for details) to generate 330 gene trees and corresponding sequence alignments with increasing number (from 0 to 10) of LGTs. Gene trees were simulated with subtree pruning and regrafting (SPR) operations from a 40-taxa rooted reference tree. Gene sequences were simulated along gene trees with variable rates of evolution among branches and sites (See *Material and Methods *for details). Maximum-likelihood (ML) trees were reconstructed using the resulting alignments. We used a different model of substitution than that used for sequence simulations to increase the chance of topological conflict resulting from reconstruction artifacts. All results of the simulation procedure are available as supplemental information (additional file 1, "simulated_dataset_prunier.zip").

We compared the performances of RIATA-HGT [11] (available in the PhyloNet package [21], EEEP [12] and Prunier based on this simulated dataset.

### Accuracy of detected transfer events

**Figure 2:**
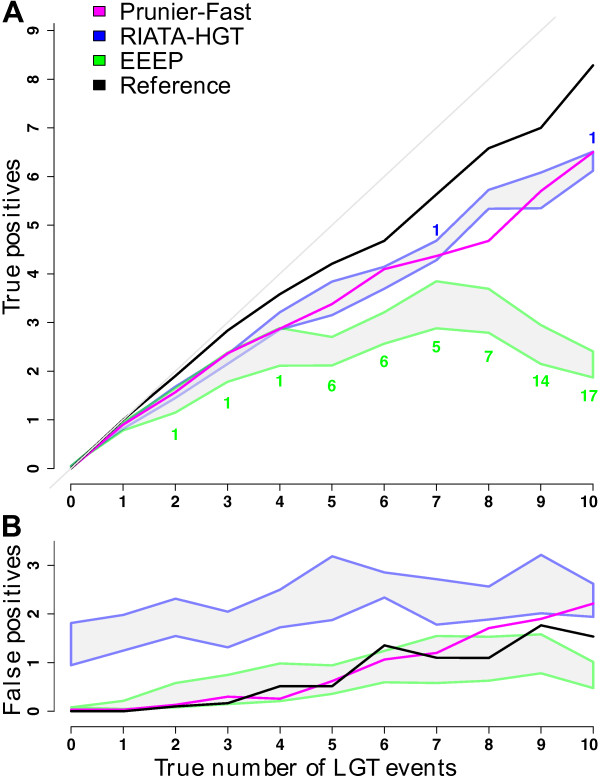
**Accuracy of transfer events detection**. A: numbers of true positive LGTs (TP). B: numbers of false positive LGTs (FP). LGT events detected in maximum-likelihood gene trees are displayed as a function of the real number of transfers per tree. Results were computed with a support threshold for topological conflict significance of 0.90. As EEEP and RIATA-HGT may propose multiple scenarios, grey areas represent the variability between the worst and the best scenarios. Areas are delimited by blue borders for RIATA-HGT and green borders for EEEP. Prunier (fast version) results are drawn in magenta. The black curves symbolize the reference boundary: those curves were obtained using RIATA-HGT (best scenario selected) on true gene trees (before sequence simulation). The green numbers below the true positives area of EEEP represent the number of unresolved cases (either quoted as "Unsolved" by the program, or resulting from a program crash). The blue numbers above RIATA-HGT TP area indicate the two cases for which the program crashed. A program crash is counted as 0 TP and 0 FP. The grey line shows a relationship 1:1 between the two axes. The estimated number of transfers by each method is the sum of the curves shown in A and B.

Rather than focusing on the accuracy of the number of detected LGTs as in previous simulation studies [10,12,11], we concentrated on the comparison of inferred scenarios with the true (simulated) one. Among the proposed LGT events, we counted the number of true and false positives (respectively TP and FP) for each method. In subsequent comparisons, all three methods used the same threshold for branch support: for EEEP, branches under this threshold are collapsed *a priori; *for RIATA-HGT, among all inferred transfers only those crossing a branch above this threshold were retained; and for Prunier, two trees were in agreement if they had no conflicting edges above this threshold. Fig. 2 shows the number of TP (fig. 2A) and FP (fig. 2B) as a function of the true number of LGT events, with a threshold of 0.90 (see additional files 2 and 3 for results with a 0.60 threshold: "TP_HGT_multi-scenar_BP60.pdf" and "FP_HGT_multi-scenar_BP60.pdf" ). For clarity, the results of another agreement function based on the ELW test [15] (Prunier-slow) which gave results very close to Prunier-fast are not presented. While Prunier gives a single scenario, both RIATA-HGT and EEEP generally propose several solutions that are equivalent in terms of number of events. We chose to show the performance of these programs by recording both the worst (with minimum TP and maximum FP) and best (i.e. with maximum TP and minimum FP) scenarios.

To obtain an idea of the best possible estimate of the number of detectable LGTs in our simulations, we ran RIATA-HGT on the true gene trees (those used for sequence simulations), and kept as a reference the best scenario proposed (see *Material and Methods *for why not all transfers are detectable). For this reference, the estimated number of transfers is quite accurate but the number of FP increases with the number of transfers, remaining under 2 (fig. 2B). The three methods have different behaviors with increasing number of transfers. RIATA-HGT performs relatively well at identifying true transfer events (fig. 2A), but consistently infers false positives, even when no transfers have been simulated (fig. 2B). Surprisingly, this number of FP does not increase significantly with the number of transfers. EEEP is very good at detecting zero or one transfer, but then increasingly fails at producing any transfer scenario, with more than 50% of failure for the 10-transfer category. Prunier is comparable to RIATA-HGT for the detection of TP (fig. 2A), but has lower rates of FP (fig. 2B). The rate of FP increases with the number of simulated transfers to reach the same level as RIATA-HGT with 10 transfers.

### Accuracy of transfer scenarios

**Figure 3:**
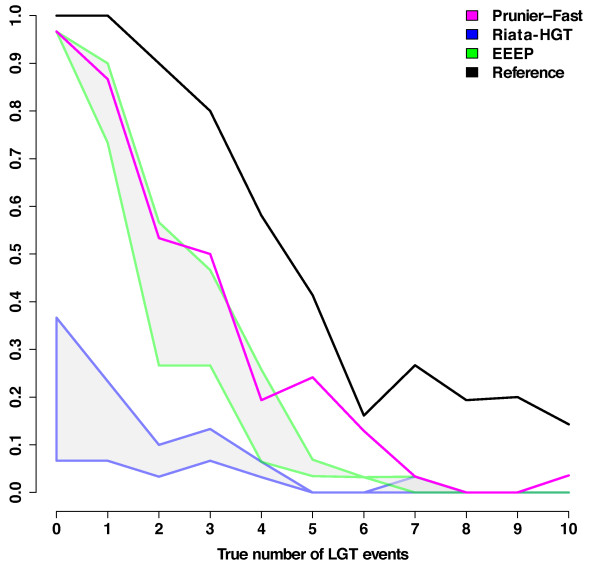
**Inference of correct complete LGT scenarios**. Proportion of correctly inferred LGT scenarios, i.e. scenarios with 100% TP and 0% FP as a function of the true number of LGT. Results were obtained with a support threshold for topological conflict significance of 0.90. As EEEP and RIATA-HGT may propose multiple scenarios, grey areas represent the variability between the worst and the best scenarios. Areas are delimited by blue borders for RIATA-HGT and green borders for EEEP. Prunier (fast version) results are plotted in magenta. The black curve symbolizes the reference boundary was obtained using RIATA-HGT (best scenario selected) on true gene trees.

Many studies trying to resolve controversial phylogenetic relationships among species have used LGT detection as a first step to retrieve sets of orthologous genes (e.g., [22-26]. In such case, it is essential that all LGT events are correctly detected in a gene family. We thus measured the proportion of gene trees in which all events of LGT were correctly identified (100% TP and 0% FP) (Fig. 3, see additional file 4 "Pc_true_LGT_events_BP60.pdf" for results with a 0.60 threshold). For Prunier and EEEP, the percentage of exact scenarios is high for low numbers of transfers, but drops relatively quickly to zero. For gene families with three transfers, these methods predict the correct scenario in about 50% of the cases. For RIATA-HGT, the proportion of correctly predicted scenarios is low even with few transfers, owing to its high rate of false positives.

### Transferred sequences

**Figure 4:**
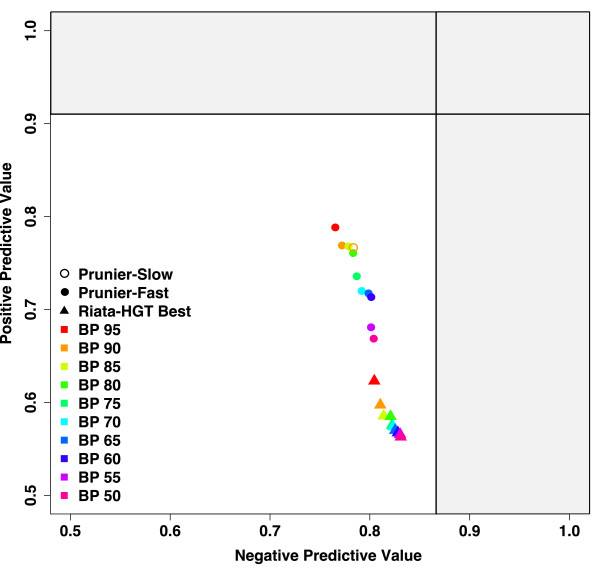
**Accuracy of transferred sequences detection**. RIATA-HGT (best scenarios selected) and Prunier (fast version) results are respectively indicated by triangles and circles. The color code corresponds to the different support values tested as a threshold for topological conflict significance. The figure represents the positive predictive value as a function of the negative predictive value (see Material and Methods) of the two methods. Black lines represent the limits given by RIATA-HGT on true trees. These statistics show how often predictions are correct when sequences are classified as transferred (PPV) or not transferred (NPV). A perfect method would have PPV = 1 and NPV = 1.

The positive predictive value (PPV) is the proportion of transferred sequences among those predicted to be transferred. The negative predictive value (NPV) is the proportion of non-transferred sequences among those predicted to be non-transferred. In this evaluation, we do not present the results for EEEP as its rate of failure with high rates of transfers precludes fair comparisons among approaches. We measured the predictive power of RIATA-HGT, selecting the best among all inferred scenarios, and Prunier for a set of simple agreement functions, defined by thresholds ranging from 0.50 to 0.95 (Fig. 4). We also tested for Prunier another agreement function which uses comparisons of the likelihood of entire tree topologies by the ELW test [15] (Prunier-slow). All values were computed on the 330 simulated gene trees.

The NPV is very similar among both methods (between 75% and 80%), and variations of the agreement function have only little effect on this value, although for all parameters, RIATA-HGT is slightly better than Prunier. In contrast, the PPV varies greatly with the threshold, especially for Prunier, but with consistently higher PPV than RIATA-HGT. These results can be summarized by computing the accuracy of the two methods, which is the proportion of correctly classified sequences (transferred or not). The accuracy ranges between 73% and 77% (mean of 74%) for RIATA-HGT, and lies between 77% and 79% (mean of 78%) for Prunier.

### Impact of the root of the species tree on reconstructed scenarios

Prunier proposes by default a scenario for every possible root of the species tree (see *Materials and Methods*). Different rootings of the species tree are expected to give different LGT scenarios, because the choice of a root constrains the clades that can be transferred. Especially, the number of transfer events is expected to be different. We examined the possibility that LGTs could inform on the true position of the root. For each of the 77 possible roots of our 40 leaves species tree, we counted the number of inferred LGTs in the 330 simulated gene families. For instance, the total number of LGTs ranged between 1370 and 1549 with a threshold of 0.90. The true root was among the two locations with the minimal number of LGT, the other one being on a neighboring edge. However, a Wilcoxon paired test showed that only 50 among the 77 possible roots were significantly different from the best root. This establishes LGT as a potential tool for rooting species trees but suggests that many gene families and relatively high rates of transfer are necessary to discern the true root.

### Application to a biological dataset

We used the dataset of Brown and colleagues [22] which contains 23 universal proteins distributed in 45 species from the three domains of life. This study pioneered a number of more recent analyses in which LGTs are searched in order to obtain a set of orthologous genes that can be concatenated to resolve a specific question [24-26]. In their article, Brown *et al*. focused on the elimination of gene transfers among domains, by manually removing those gene families that supported a non-monophyletic bacterial domain. Nine gene families were removed on this criterion, which reduced the dataset to 14 genes. Two different species trees were reconstructed: a first one based on the whole dataset (23 genes), which was deemed artifactual due to LGT and a second one based on the cleansed dataset (14 genes). The two trees mainly disagreed on the position of the early diverging bacterial phyla, respectively spirochaetes and hyperthermophiles.

Although detecting transferred genes with a certain reference tree and using the remaining sequences to infer a tree would be a circular reasoning, it is possible to use our algorithm to test the hypothesis that a dataset is devoid of LGT. We ran Prunier (slow and fast version with a threshold of 0.80 and 0.90) using both trees as a reference, and looked at the number of transfers inferred in the 23 genes. The simulation results showed very similar results for the slow and fast version of Prunier (for example, the number of detected transfers by the slow and fast methods and the threshold of 0.80 were correlated with R = 0.97). With real data, we also observed a high correlation among different agreement methods (the best being between slow and fast_0.80: R = 0.63) but with marked differences for some gene families. For instance the initiation factor 2 (IF-2) gene tree is found to be completely congruent with the 23-gene reference tree using the slow test, while the fast version infers 9 transfers. Reciprocally, some other genes showed higher transfer rates with the slow test, for instance ribosomal protein S11 where 13 (23-gene tree) or 11 (14-gene tree) LGTs were detected with the slow version versus one with the fast version, for both reference trees. The mean number of transfers invoked in the two sets of genes identified by Brown *et al*. is significantly different, for the fast version, regardless of the reference tree (Wilcoxon test between numbers of transfer events for each tree shown in Table 1: p-value = 0.04 for both fast_0.80 and fast_0.90 with the 23-gene tree, p-value = 0.004 and 0.03 with the 14-gene tree for fast_0.80 and fast_0.90, respectively). This suggests that the manual criterion originally used by Brown *et al*. [22] for gene exclusion correctly identified genes with high transfer rates. Nevertheless, many among the 14 genes retained in the second dataset show a significant amount of transfers with our method. This raises the question of whether the combination of such a limited amount of genes with such a strong degree of conflict can really yield a reliable species tree.

**Table 1:** Analysis of 23 universal gene families with Prunier with two reference trees

Protein	Alignment length	LGT inferred by Prunier (slow and fast version)					
		
(* indicates LGT diagnosis by Brown et al. )		23 genes tree reference tree			14 genes tree reference tree		
		
		Slow	Fast-80	Fast-90	Slow	Fast-80	Fast-90
aspartyl-tRNA synthetase	249	4	8	8	6	7	7

glutamyl-tRNA synthetase	188	12	12	12	13	11	11

leucyl-tRNA synthetase	358	9	11	11	11	13	13

initiation factor 2	337	8	9	0	3	3	1

elongation factor G	536	10	11	5	11	9	4

elongation factor Tu	340	0	9	9	7	9	9

ribosomal protein L2	192	2	13	1	6	8	0

ribosomal protein S5	131	7	2	2	4	2	2

ribosomal protein S8	118	7	5	0	7	5	0

ribosomal protein S11	110	13	1	1	11	1	1

DNA-directed RNA polymerase β chain	537	0	8	2	8	7	2

DNA topoisomerase I	236	10	13	4	8	12	3

DNA polymerase III subunit	194	3	2	1	2	2	0

signal recognition particle protein	298	3	8	1	4	5	1

alanyl-tRNA synthetase (*)	502	5	7	3	4	11	3

histidyl-tRNA synthetase (*)	166	7	18	9	14	15	11

isoleucyl-tRNA synthetase (*)	552	8	9	7	10	9	8

methionyl-tRNA synthetase (*)	306	11	9	7	0	12	10

phenylalanyl-tRNA synthetase β subunit (*)	177	7	11	3	6	11	3

threonyl-tRNA synthetase (*)	305	13	16	16	11	16	16

valyl-tRNA synthetase (*)	538	9	12	12	7	9	8

aminopeptidase P (*)	95	19	21	21	24	21	21

rRNA dimethylase (*)	126	11	13	2	8	11	2

## Discussion

Detecting LGTs using phylogenetic approaches is a challenge for several reasons. First, the reconstruction of optimal scenarios explaining the discrepancies between two trees is a complex algorithmic problem. Second, in practice, not all of these discrepancies require a biological explanation, because reconstructed gene trees are imperfect representations of a true gene history. We aimed at testing approaches that propose reconciliation scenarios in the typical situation faced by biologists, *i.e.*, the comparison of an unrooted gene tree reconstructed from sequences with a rooted reference. Two published methods, EEEP and RIATA-HGT corresponded to these criteria. We propose here a new algorithm that applies to this situation, along with an evaluation of its performance in comparison to EEEP and RIATA-HGT on a simulated dataset.

### Simulating LGT

It is difficult to simulate datasets that compare to real ones, in particular in terms of the phylogenetic artifacts they produce. We used a procedure [20] that creates SPR movements on a rooted reference tree and then simulates different evolutionary rates among branches of the tree and sequence sites, attempting to produce as realistic as possible gene alignments. We then reconstructed ML trees with a model of sequence evolution different from the one used for sequence simulation.

The results of our simulations show that working on reconstructed gene trees poses a substantial problem to LGT detection methods. This is evident from the comparison of the result of RIATA-HGT when used on simulated *vs*. reconstructed gene trees (Reference *vs*. RIATA-HGT results in Fig. 2 to 4). The best scenario among those provided by RIATA-HGT on the simulated gene trees (without sequence simulation) was used as a measure of the best scenario inferable. According to this reference, most transfers (>80%) are detectable and the expected amount of false positives is relatively low (Fig. 2B).

All methods were able to correctly identify simulated LGT events to a certain extent. EEEP appeared quite accurate when proposing a solution, but its frequency of failure (that is, the program stops without LGT scenario output) makes it difficult to use with high levels of transfers. In contrast, the two other programs almost always gave a result and generally produced reasonably good solutions, even with complex transfer scenarios. However, RIATA-HGT is characterized by high rates of false positives which explain a consistently low proportion of exact RIATA-HGT scenarios even with few LGT events (Fig. 3).

### A new approach for transfer detection driven by statistical criterion

Our new approach, Prunier, sequentially cuts branches that are found to generate significant conflict among trees. We reasoned that a fast way to reach agreement between trees is to first cut those branches that are responsible for the highest part of the conflict. Accordingly, Prunier relies on a ranking of branches that are common to the two trees based on the amount of conflict that is removed when the branch is cut. In the current implementation, this amount of conflict is a function that depends on the statistical support of internal branches in the gene tree (see *Material and Methods*) and the branch with the highest rank is cut first. The algorithm is thus directed by statistical information provided by branch support in the gene tree and estimating the statistical support of branches is a prerequisite of the application of Prunier. This contrasts with EEEP and RIATA-HGT, where branch support is only used to discard some irrelevant groupings. These approaches rely on combinatorial algorithms that search to enumerate all topological solutions. In contrast, Prunier uses statistical information in the gene tree to guide its search and avoid non significant transfer events to be invoked. As a result, RIATA-HGT and EEEP usually propose a set of scenarios that are equivalent in terms of number of transfers, when Prunier always proposes a unique scenario. Although providing a single solution is not necessarily an advantage, the scenarios found by Prunier are always better than the best among those proposed by RIATA-HGT, as Prunier consistently infers fewer false positives for equivalent number of true positives. In comparison with EEEP, the algorithmic shortcut used by Prunier does not seem to alter the performance in terms of quality of the results but appears as a gain in efficiency as Prunier always terminates. We can presume that the type of statistics used by Prunier for branch support is critical for its performance. The LR-ELW values computed by the Treefinder program [19] seem to give good results, at least with simulated datasets, but others like bootstrap could also be tested.

### Program parameters: threshold and agreement function

Concerning the choice of a support threshold in the fast version, it is important to tune this parameter according to user needs. Higher support value thresholds should be favored when seeking transfer events with high confidence. In contrast, lower thresholds should be preferred when trying to identify orthologous sequence sets. However, the negative predictive value (NPV) does not vary a lot when decreasing the threshold, whereas the positive predictive value (PPV) drops quickly (Fig. 4). This suggests that higher threshold values are a relatively good choice in all situations. When comparing Prunier in its fast and slow versions on simulated data, it appeared that using the maximum-likelihood test ELW as an agreement function instead of a function based on branch support values did not improve the quality of transfer detection (Fig. 4), and that the resulting LGT scenarios are strongly correlated. In this case, it does not seem beneficial to use such a computationally intensive method in Prunier. However, in contrast with simulated results, the fast and slow implementations of Prunier gave correlated but sometimes contrasting estimates of LGT numbers on a real dataset (Table 1). It is difficult to argue for the use of "slow" *vs*. "fast" agreement functions on such data. However, all methods detected a large number of transfers in most gene families, including those conserved for concatenation. This means that there remains a strong phylogenetic conflict among concatenated genes. Interestingly, tRNA synthetases, which have been reported as prone to transfer, yield particularly high numbers of LGTs.

### Rooting the species tree

One particular feature of Prunier is its ability to search efficiently for scenarios of LGT using an unrooted reference tree, thereby proposing different solutions for different possible roots. Interestingly, different roots in the species tree yield different LGT scenarios, and an overall score could be computed on all 330 gene trees. We showed that the best rooted tree (minimum number of LGTs) is the true root of the reference tree. This suggests that non-optimal roots tend to produce more LGTs than the true one. This echoes with previous reports that LGT events, when seen as a shared character, could sometimes provide phylogenetic information [27]. We demonstrate here that Prunier provides information for rooting the reference tree, since the true root position was the best (with high support threshold value) or among the best (for lower threshold values, data not shown) in terms of LGT number. Although the number of gene trees necessary to unambiguously root a reference tree is probably high, the LGT criterion could be used to exclude potential roots.

## Conclusions

We propose a new method, Prunier, based on the statistical reconciliation of a gene tree and a reference tree by searching for the maximum statistical agreement forest. We compared Prunier and two other programs, EEEP [12] and RIATA-HGT [11] on a simulated dataset attempting to reflect realistic conditions of gene families analyses. Prunier performance and robustness demonstrates its appeal over other tested methods. It proposes a unique scenario of LGTs that compares to the best selected scenarios of the two other methods, produces fewer false positives, in particular compared to RIATA-HGT, and is able to infer true transfer events even for the most complex gene histories, what EEEP fails to do. Being fast and accurate, Prunier can be used to study phylogenomic datasets.

## Methods

### Implementation and availability of Prunier

Prunier is implemented in C++ language, using the Bio++ library [28]. Maximum-likelihood (ML) estimation of branch lengths and trees, and ELW tests are performed by Treefinder [19]. It is available at: http://pbil.univ-lyon1.fr/software/prunier

### Dealing with possible rootings

We have presented the MSAF problem with a rooted species tree and an unrooted gene tree as input. However, the exact position of the root of the species tree is often not known. A solution would be to try every root, and give as many scenarios as there are branches in *T*_*S*_. But many root positions are equivalent in the sense that an LGT scenario for a given root is also valid for all roots that are not included in a transferred subtree. We developed a way to explore more efficiently a set of root positions. Prunier can take two unrooted trees as input, and output several statistical agreement forest (SAF) decompositions, one for each set of equivalent roots.

Let *R *be a subset of edges of *T*_*S*_, denoting the possible root locations. *R*, *T*_*S *_and *T*_*G *_are sets of edges, so set operations ∩ or \ are used. At first, *R *might be either the set of all edges in *T*_*S*_, if no information is available on the root position in the species tree, or any subset of branches given by the user. A non conflicting edge *e *of *T*_*S *_cuts *T*_*S *_into two disjoint subtrees *T*_*S*_(*S*_*i*_) and *T*_*S*_(*S*_*j*_). *S*_*j *_is a possible common clade if *R*is not fully included in *T*_*S*_(*S*_*i*_). For each possible common clade *S*_*i *_a conflict score is computed if *T*_*G*_(*S*_*i*_) statistically agrees with *T*_*S*_(*S*_*i*_) and the top scoring *S*_*i *_is selected. If for the top scoring *S*_*i*_, (*T*_*S*_(*S*_*i *_∩*R*) = Ø, *S*_*i *_is considered as a group of transferred species and is removed from *T*_*S *_and *T*_*G*_. If (*T*_*S*_(*S*_*i*_) ∩ *R*) = Ø, the two possible positions of the root, *R *∩ *T*_*S*_(*S*_*i*_) and *R *∩*T*_*S*_(*S*_*j*_), are examined: either the root is not in *S*_*i *_and *S*_*i *_is considered as a group of transferred species or the root is in *S*_*i *_and the next top scoring common clade is searched with *R *= *R *∩ *T*_*S*_(*S*_*i*_). It results in a bifurcating procedure where the number of explored scenarios is lower than the number of possible roots of *T*_*S*_. The procedure to approach the MSAF can be described as follows:

Input: two unrooted trees *T*_*S *_and *T*_*G*_, and a subset *R *of edges of *T*_*S *_where the root may lie: {*T*_*S*_, *T*_*G*_, *R*}.

Output: a decomposition in a SAF for each root position contained in *R.*

• If *T*_*S *_and *T*_*G *_disagree:

▪ Find the common clade *S*_*i*_, such that *T*_*S *_(*S*_*i*_) and *T*_*G *_(*S*_*i*_) agree, with maximum conflict score

▪ If (*T*_*S*_(*S*_*i*_) ∩ *R*) ≠ Ø then

Recursively run the program with input {*T*_*S*_, *T*_*G*_, *T*_*S*_(*S*_*i*_) ∩ *R*}

// 1^st ^hypothesis on rooting: *T*_s _(*S*_*i*_) contains the root, we choose another set of species *S*_*i*_

▪ Recursively run the program with input {*T*_*S*_\*T*_*S*_(*S*_*i*_), *T*_*G*_\*T*_*G*_(*S*_*i*_), *R*\*T*_*s*_(*S*_*i*_)}, and add *S*_*i *_to the SAF

// 2^nd ^hypothesis on rooting: *T*_*S*_(*S*_*i*_) does not contain the root, we prune it

• For each possible root of the initial set *R*, output the SAF.

### Definition of the Agreement function

A MSAF of a species tree and a gene tree has two types of components: a backbone tree where the root of the species tree lies, which is interpreted as the set of non transferred sequences, and as many subtrees as inferred transfers. The backbone tree in *T*_*G *_contains the root but without information on its exact position. In contrast, all transferred subtrees are rooted by the backbone in both *T*_*S *_and *T*_*G *_. An appropriate agreement function must hence handle both rooted (transferred) and unrooted (backbone) trees.

The method can potentially handle any agreement function. We implemented two variants, respectively called "slow" and "fast" versions.

- A "slow" implementation that depends on the ELW test with a default p-value of 0.05 [15]. Because ELW is an unrooted test, our agreement function first checks for the presence of a well supported bipartition in conflict with the position of the root. The threshold for this additional criterion is set by default at 0.90.

- A "fast" implementation where two (rooted or unrooted) trees are said to be in agreement if the two trees do not contain any supported conflicting edge according to a user defined threshold. This test handles both rooted and unrooted trees.

### Scoring candidate transfers

For each edge *e *of the tree, we have a support value *SV*(*e*). In our implementation, we use the LR-ELW (local rearrangement ELW) support values given by Treefinder [19], but any other statistics can be used.

For each clade *S*_*i*_, let *e*_*1*_,...,*e*_*n *_be the conflicting edges of *T*_*G *_and *T*_*S *_that are no longer conflicting in *T*_*G*_\*S*_*i*_and *T*_*S*_\*S*_*i*_.The score of *S *_*i *_is defined by:(1)

This value is meant to assess the amount of conflict the group *S *_*i *_is responsible for. The power law, empirically set to the value of 5, is a way of favoring groups removing highly supported conflicting bipartitions.

### Simulations of LGT

We used the program described by Galtier [20] to simulate LGT and sequence alignments. This program starts from a rooted species tree and modifies it to introduce evolutionary rate variations between species. Then gene trees are generated from the reference trees. Transfers are created by subtree pruning and regrafting (SPR operations) of random clades, where donors and acceptors are drawn according to a Poisson law. Time constraints are given by branch lengths, allowing transfers to occur only between contemporary lineages. Variations of evolutionary rate among genes (diameter of the tree), and between lineages are finally introduced. Then artificial sequences are simulated along each gene tree, under a given model of substitution. Evolution of sequences also includes random substitution rate variations between sites.

We simulated a dataset based on a 40-taxon species tree, with a uniform gene length distribution between 100 and 400 amino-acids, under a JTT model of substitution [29]. Number of transfers increased from 0 to 10. We simulated 30 alignments and trees per number of gene transfers, resulting in a final dataset of 330 simulations.

In order to increase the level of difficulty of our simulated dataset, and thus come closer to real biological datasets, maximum-likelihood (ML) gene trees were reconstructed with Treefinder [19] (WAG [30] + G8 + I + F) from the simulated gene alignments. The simulated dataset can be downloaded at http://pbil.univ-lyon1.fr/software/prunier/simulated_dataset_prunier/ or found in the additional file 1: "simulated_dataset_prunier.zip".

### Dealing with invisible LGT

Certain simulated transfers have no impact on the phylogeny, and thus are phylogenetically undetectable, even if they are biologically sound. A transfer between sister taxa, or between a father and his son will not provoke any topological conflict. These transfers were filtered out, in order to quantify how many transfer events, among those detectable, have been correctly detected. Nevertheless, one has to keep in mind that these transfers might exist, and that the number of detected LGTs is therefore under-estimated by phylogenetic methods of transfer detection. Multiple transfers can also be indistinguishable when ancestors of sister groups independently received gene from a same species. For example if two sister groups receive a gene from the same donor, a detection based on topology discrepancies would infer one LGT instead of two. These transfers were not sorted out and some instances of such transfers can be present in the dataset. This is why we used a topological reconciliation of simulated gene trees before sequence simulation to roughly estimate the amount of detectable transfers.

### Benchmark on simulations: estimating the performance of LGT detection methods

Different statistics were used to evaluate the performance of all LGT detection programs. For transfer events, we calculated the number of true and false positives (TP and FP). It was not possible to compute a number of true and false negatives (TN and FN) for transfer events because sequences that are not cut out of the tree do not correspond to a single event. Therefore, we also defined TP, FP, TN and FN for leaves of the tree that were either correctly or incorrectly cut out by an inferred transfer event. This allowed us to compute a sensitivity (true positive rate), specificity, positive and negative predictive values (PPV the precision, and NPV respectively), and accuracy (P standing for positives, N standing for negatives) such that:(2)

### Comparing root scenarios

In order to compare scenarios of transfers between all possible roots, we performed all possible paired-Wilcoxon tests between number of transfers per gene families, and applied to the computed p-values a false discovery rate correction for multiple comparisons [31].

### Biological dataset

We used the dataset of Brown and colleagues [22] (Table 1). This dataset gathers 23 universal proteins, present in 45 species of the three kingdoms of life. These proteins are mainly part of the translational apparatus (tRNA synthetases and ribosomal proteins). The others play a role in transcription, replication or other basal metabolic functions. We ran Prunier (slow and fast versions, the latter with two different support value thresholds: 0.80 and 0.90) on these genes, using both reference trees proposed by Brown *et al*.: the initial 23-gene tree, and the 14-gene tree obtained on a cleansed set of genes, after the removal of 9 genes suspected of lateral gene transfer on the basis of the non-monophyly of the bacterial domain.

## Availability and requirements

• **Project name: **Prunier

• **Project home page: **http://pbil.univ-lyon1.fr/software/prunier

• **Operating systems: **Linux and Mac OS

• **Programming language: **C++ (Bio++ library: http://kimura.univ-montp2.fr/BioPP/ )

• **Other requirements: **Treefinder: http://www.treefinder.de/

• **License: **freeware

• **Any restrictions to use by non-academics: **none

## Abbreviations

LGT: Lateral Gene Transfer; ML: Maximum likelihood; ELW: Expected likelihood weights; LR: Local rearrangement; MAF: Maximum agreement forest; SAF: Statistical agreement forest; MSAF: Maximum statistical agreement forest; PPV: Positive predictive value; NPV: Negative predictive value; FP: False positives; TP: True positives; FN: False negatives; TN: True negatives

## Authors' contributions

SSA, ET, MG and VD designed the algorithm and the simulation procedure. SSA implemented the program and conducted the experiments. SSA, ET, MG and VD wrote the paper. All authors read and approved the final version of this manuscript.

## Supplementary Material

Additional file 1 **The 330 simulated alignments and corresponding gene trees (true and ML) along with the 40-taxa reference species tree**. Click here for file

Additional file 2 **True positives (transfer events) with a 0.60 threshold for EEEP, Prunier and RIATA-HGT.** For detailed legend see Fig. 2. Click here for file

Additional file 3 **False positives (transfer events) with a 0.60 threshold for EEEP, Prunier and RIATA-HGT.** For detailed legend see Fig. 2. Click here for file

Additional file 4 **Proportion of correct complete scenarios with a 0.60 threshold for EEEP, Prunier and RIATA-HGT**. For detailed legend see Fig. 3. Click here for file
