# Supplementary figures and images for: Detecting lateral gene transfers by statistical reconciliation of phylogenetic forests
Source: BMC Bioinformatics. 2010 Jun 15;11:324. doi: 10.1186/1471-2105-11-324 (PMC2905365; doi:10.1186/1471-2105-11-324)

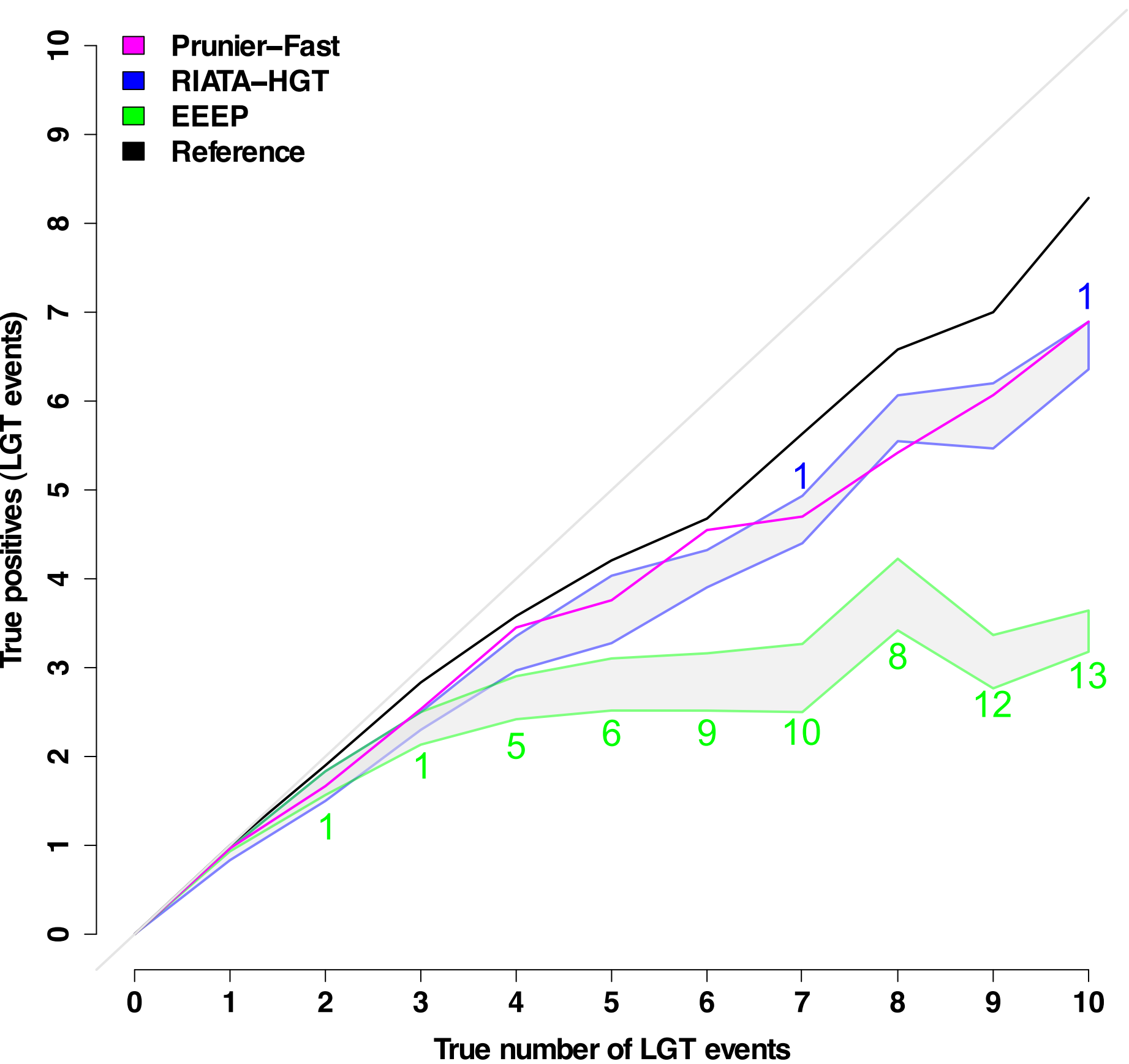

Supplement: Additional file 2 — True positives (transfer events) with a 0.60 threshold for EEEP, Prunier and RIATA-HGT. For detailed legend see Fig. 2. [file 1471-2105-11-324-S2.PDF]

# Accuracy of LGT events detection

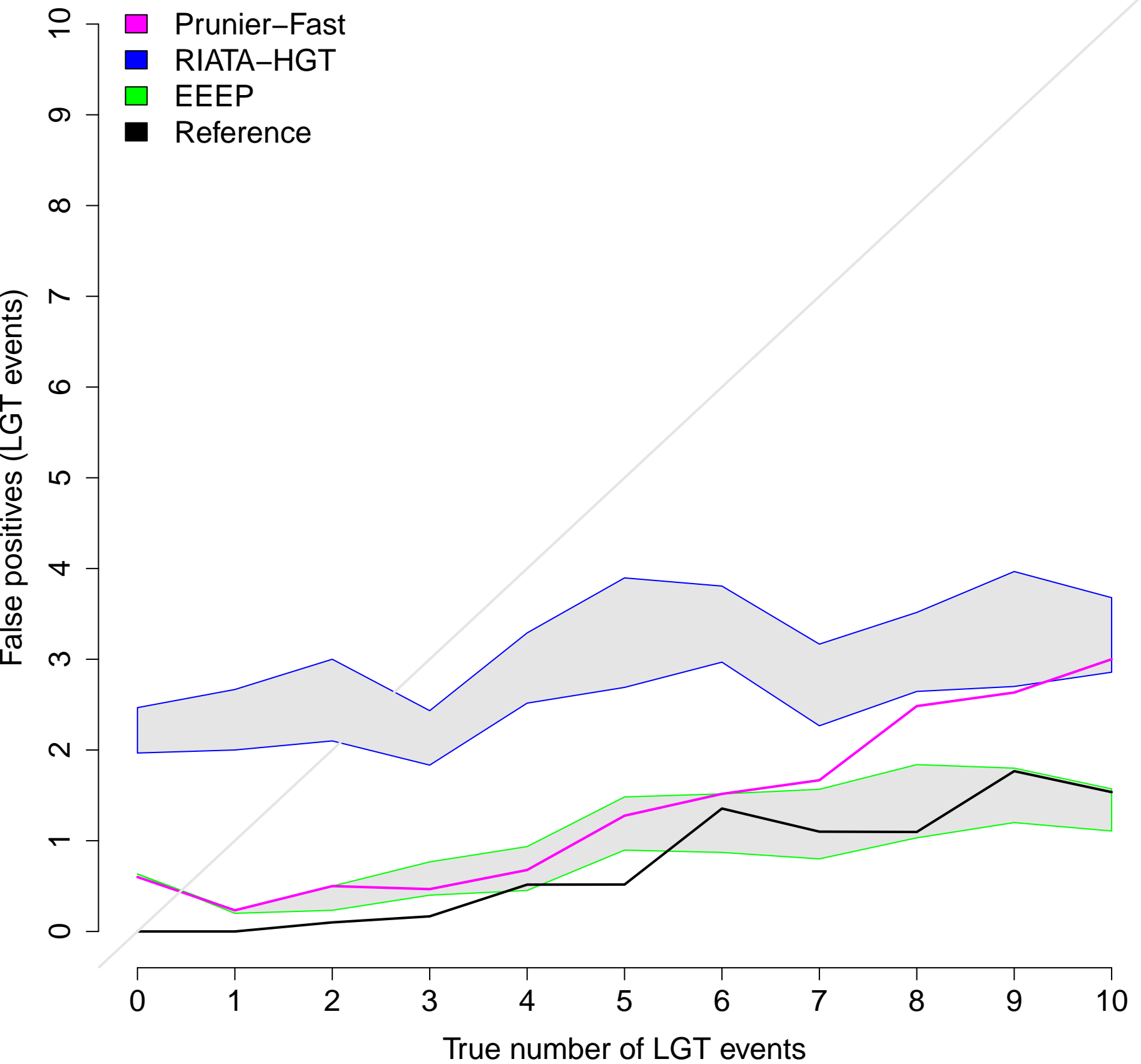

Supplement: Additional file 3 — False positives (transfer events) with a 0.60 threshold for EEEP, Prunier and RIATA-HGT. For detailed legend see Fig. 2. [file 1471-2105-11-324-S3.PDF]

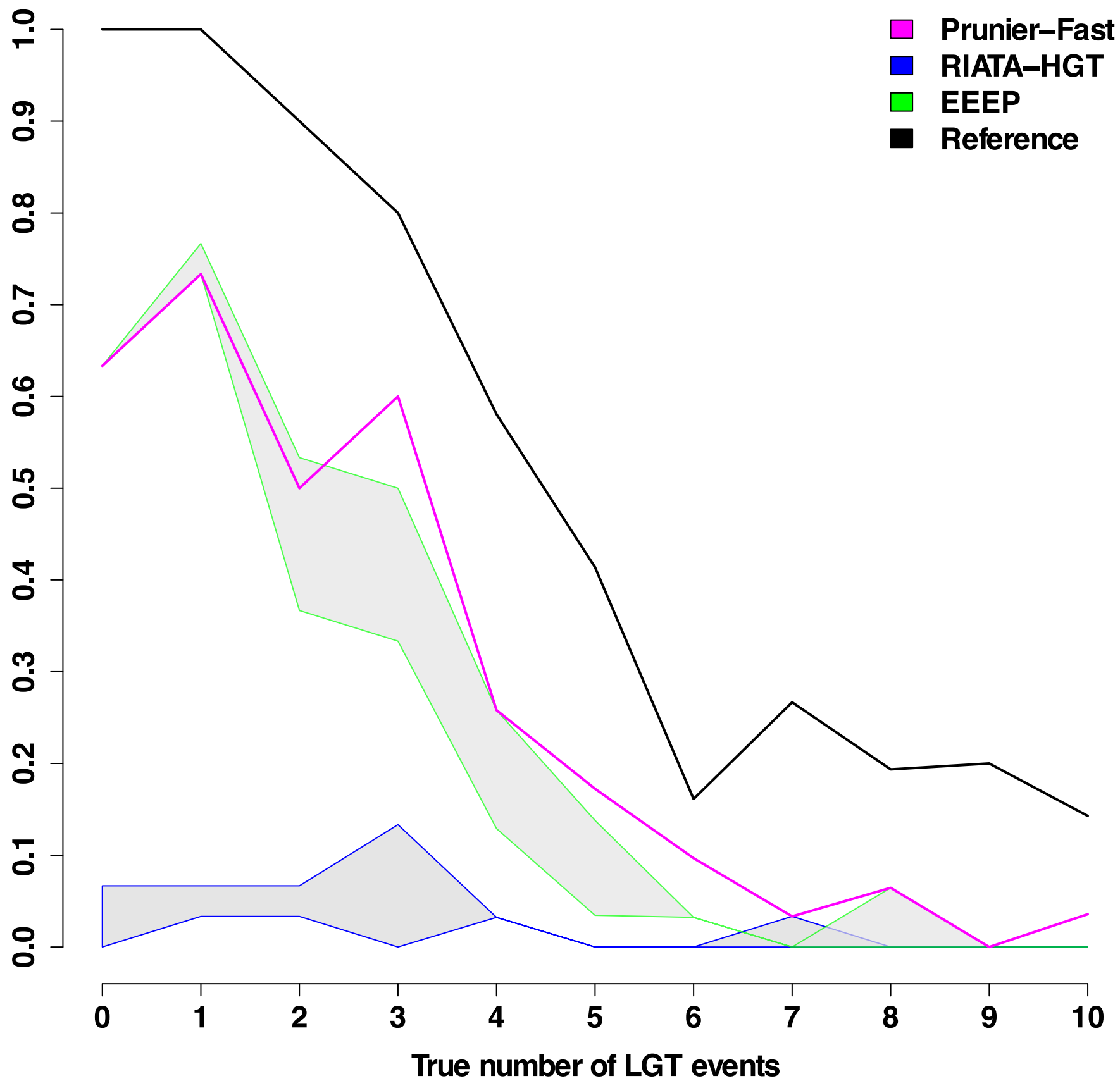

Supplement: Additional file 4 — Proportion of correct complete scenarios with a 0.60 threshold for EEEP, Prunier and RIATA-HGT. For detailed legend see Fig. 3. [file 1471-2105-11-324-S4.PDF]
